# Pharmacokinetic Disposition of Amiodarone When Given with an Intralipid Rescue Strategy

**DOI:** 10.3390/pharmaceutics13040539

**Published:** 2021-04-13

**Authors:** Sean N. Avedissian, Michelle Pham, Medha D. Joshi, Marc H. Scheetz, Ashkan Salamatipour, Jeffin M. Panickar, Khrystyna Hlukhenka, Cristina Miglis, Athanasios Chalkias, Theodoros Xanthos

**Affiliations:** 1 Antiviral Pharmacology Laboratory, Center for Drug Discovery, University of Nebraska Medical Center (UNMC), Omaha, NE 68198, USA; michelle.pham@unmc.edu; 2 Department of Pharmacy Practice and Sciences, College of Pharmacy, University of Nebraska Medical Center, Omaha, NE 68198, USA; 3 Department of Pharmaceutical Sciences, College of Pharmacy-Glendale, Midwestern University, Glendale, AZ 85308, USA; mjoshi@midwestern.edu; 4 Department of Pharmacy Practice, Midwestern University College of Pharmacy, Downers Grove, IL 60515, USA; mschee@midwestern.edu; 5 Pharmacometric Center of Excellence, Midwestern University College of Pharmacy, Downers Grove, IL 60515, USA; 6 Department of Pharmacology, College of Graduate Studies, Midwestern University, Downers Grove, IL 60515, USA; 7 Department of Pharmacy, Northwestern Medicine, Chicago, IL 60611, USA; 8 Department of Pharmaceutical Sciences, Midwestern University College of Pharmacy, Downers Grove, IL 60615, USA; asalamatipour84@midwestern.edu (A.S.); jpanickar67@midwestern.edu (J.M.P.); khlukhenka87@midwestern.edu (K.H.); cristina.miglis@fda.hhs.gov (C.M.); 9 Department of Anesthesiology, Faculty of Medicine, University of Thessaly, 41500 Larisa, Greece; athchalkias@uth.gr; 10 Department of Physiology and Pathophysiology, School of Medicine, European University Cyprus, Nicosia 1516, Cyprus; T.Xanthos@euc.ac.cy

**Keywords:** amiodarone, pig, HPLC, Intralipid, pharmacokinetic

## Abstract

While the antiarrhythmic drug amiodarone is commonly used in clinical practice, it has a narrow therapeutic index that can lead to acute overdose. One proposed method to deal with this toxicity is lipid emulsion therapy, which may potentially quench the free amiodarone in blood and prevent its further distribution to target organs and tissues. In this study, we utilize an established swine model to examine the effects of Intralipid™ (IL) administration for acute amiodarone toxicity. A total of 14 pigs received an overdose of intravenous amiodarone. After twenty minutes, half of the pigs (*n* = 7) received IL while the control group (*n* = 7) received normal saline. Serum concentrations of amiodarone were then analyzed using a validated high-performance liquid chromatography (HPLC) method. Noncompartmental pharmacokinetic analyses were performed on the observed concentrations. There were no statistical differences in the area under the concentration time curve (6 h) or clearance, but there was a difference in the half-life between the two groups (3.12 vs. 0.85 h, *p* = 0.01). The administration of IL did not statistically change the overall exposure of amiodarone in the blood in the first 6 h; however, trends toward prolonged blood retention in the IL group were seen.

## 1. Introduction

Amiodarone is a highly effective antiarrhythmic agent used to treat a variety of supraventricular and ventricular cardiac arrhythmias [1]. It is categorized as a class III antiarrhythmic, but it has electrophysiologic characteristics of all four Vaughan–Williams classifications [2]. This unique mechanism of action has greatly enhanced its clinical utility; however, its use is often complicated by unusual pharmacokinetics and unfavorable adverse effects.

Amiodarone’s adverse effects can be divided into two categories: those which occur during the intravenous loading dose and those associated with chronic oral administration [3]. This hypotensive effect has been observed when amiodarone is given intravenously in atrial fibrillation and is especially pronounced when administered as a concentrated intravenous dose in shock-resistant shockable rhythms [4,5].

In humans, amiodarone widely distributes in body tissues owing to its large volume of distribution (66 L/kg), and it is associated with a very long terminal half-life (40–55 days) [6,7]. However, pharmacokinetics in the proximal infusion period are more complex and largely explained by drug transit from the serum to peripheral and deep tissue stores [8]. The relationship between amiodarone serum concentrations and adverse events during acute infusion is not well established, although side effects have been noted at concentrations above 2.5 µg/mL [9]. In addition, the distribution of amiodarone and its principal active metabolite, desethylamiodarone, varies depending on the dose and route of administration [10]. Like amiodarone, desethylamiodarone is highly lipophilic and has been noted to accumulate in peripheral compartments and adipose tissue, specifically with chronic oral administration. It has been reported that amiodarone is mainly metabolized to desethylamiodarone by the cytochrome P450 isoenzyme; however, this conversion is minimal when it is given as an acute intravenous infusion. For this reason, amiodarone toxicity associated with intravenous use is thought to be caused primarily by the parent compound [8]. While intravenous amiodarone has been shown to cause hypotension in humans and swine, limited clinical data exist on the treatment of acute toxicity [5,11].

One proposed method to reverse amiodarone toxicity is utilization of a lipid emulsion that sequesters excess drug in the serum or target tissues. However, only two studies have evaluated the effectiveness of this method to date, and they found that lipid infusion attenuated the effects of amiodarone in swine models [12,13]. The first of these studies found that simultaneous administration of amiodarone with a lipid infusion successfully counteracted the hypotensive effects of intravenous amiodarone infusion in swine [12]. The clinical utility of lipid emulsion sequestration was then evaluated in a subsequent swine model. This study administered intravenous lipid emulsion after induction of acute amiodarone toxicity and found that administration of the lipid emulsion attenuated the hypotensive effects of amiodarone toxicity for a period of 15 min compared to animals receiving normal saline [13].

Consequently, in this study, we used the swine model to examine the sequestration effects of Intralipid™ (IL) administration after acute amiodarone overdose and looked for pharmacokinetic changes due to lipid infusion. We analyzed changes in serum concentrations of amiodarone by adapting a previously published HPLC method [14].

## 2. Materials and Methods

### 2.1. Porcine Model

Animal model ethics statement: The protocol was approved by the Protocol Evaluation Committee of the ELPEN Experimental Research Center according to Greek legislation regarding ethical and experimental procedures. Reference number 7436/17-11-2014. Female Landrace–Large/White swine from experiments in [13] had samples analyzed pharmacokinetically in this study.

Brief, 14 female Landrace–Large/White piglets aged 10–15 weeks, with average weight 19 ± 2 kg, all from the same breeder (Validakis, Athens, Greece), were studied. All animals were prepared in a standardized fashion in the research facility (ELPEN Experimental Research Center, Pikermi, Greece). Initial sedation was achieved by intramuscular administration of ketamine hydrochloride (10 mg/kg), midazolam (0.5 mg/kg), and atropine (0.05 mg/kg). Induction of anesthesia was achieved with an intravenous bolus dose of propofol (Diprivan 1% *w*/*v*; AstraZeneca, Luton, UK) (2 mg/kg) and fentanyl (Janssen Pharmaceutica, Beerse, Belgium) (2 μg/kg) via the marginal auricular vein [11,15]. The pigs were intubated with a 4.5 mm cuffed endotracheal tube and mechanically ventilated with a volume-controlled ventilator with tidal volume 10 mL/kg and FiO2 0.21. End-tidal CO2 (ETCO2) was monitored by in-line waveform capnography (Tonocap TC-200-22-01, Engstrom Division Instrumentarium Corp., Helsinki, Finland), and the respiratory frequency was adjusted to maintain ETCO2 between 35 and 40 mm Hg. Anesthesia was maintained with continuous infusion of 150 μg/kg/min propofol, 0.6 μg/kg/min fentanyl, and 20 μg/kg/min cisatracurium. Cardiac rhythm and heart rate were monitored by electrocardiography (ECG), using leads I, II, III, aVR, aVL, and aVF, while pulse oximetry was monitored continuously. For measurement of aortic pressure, an arterial catheter (model 6523, USCI CR, Bart, Papapostolou) was inserted and forwarded into the descending aorta after surgical preparation of the right internal carotid artery. The systolic and diastolic pressures were recorded, whereas mean arterial pressure was determined by the electronic integration of the aortic blood pressure waveform [15]. A catheter was inserted into the right internal jugular vein for continuous measurement of central venous pressure, while the left internal jugular vein was also cannulated for blood sampling. Intravascular catheters were attached to pressure transducers that were aligned to the level of the right atrium and were calibrated before use.

After allowing the animals to stabilize for 60 min, amiodarone overdose (1 mg/kg/min) was initiated for a maximum of 20 min, or until the mean arterial pressure (MAP) reached 50% of the baseline value, or the heart rate became less than 50 bpm. The animals were then randomized into 2 groups. Group A (*n* = 7) received 0.9% saline solution and Group B (*n* = 7) received 20% IL. The IL used is FDA-approved for human usage [16]. A bolus dose of 2 mL/kg over 2 min was initially administered in both groups, followed by a 45 min infusion (0.2 mL/kg/min) in either arm of the study. All animals were fully monitored for 4 h and, after the end of the experiment, they were humanely euthanized by an intravenous overdose of pentobarbital 3 g. Pharmacokinetic blood samples were obtained at the following times in minutes: 0, 5, 10, 15, 20, 22, 35, 50, 65, 80, 140, 200, 260.

### 2.2. Chemicals and Reagents (Drug Assay)

Amiodarone (USP) was obtained commercially (Enzo Life Science, CA, USA). Tamoxifen (Alfa Aesar, Ward Hill, MA, USA), acetonitrile, and methanol were purchased from VWR International (Radnor, PA, USA). Formic acid was obtained from Sigma-Aldrich (Atlanta, GA, USA). All solvents used were of HPLC or LC/MS/MS grade. Purified porcine serum from animals residing in the United States that were less than 12 months of age was obtained from Rocky Mountain Biologicals (Missoula, MT, USA). An acetonitrile: methanol: 50 mM phosphate buffer with 0.1% formic acid at pH 3.1 (55:10:35) was used for dilution.

### 2.3. HPLC Method

An Agilent 1260 series HPLC system (Agilent Technologies, Waldbronn, Germany) equipped with a quaternary pump and temperature-controlled auto-sampler tray maintained at 4 °C was used. Instrumental control and chromatographic data acquisition were performed with Agilent ChemStation Rev. B.03.01 software (Waldbronn, Germany). A reversed-phase column (LiChroCART Purospher Star; C18; 55 × 4 mm; 3 mm particle size) purchased from Merck KGaA, protected by a Security Guard Column, KrudKatcher guard column (Phenomenex, Torrance, CA, USA), was used. Column temperature was maintained at 25 °C. An isocratic method was used to separate the analyte, with flow rates set at 0.25 mL/min. Eluents were scanned at multiple wavelengths such as 198, 230, 240, and 254 nm; however, a wavelength of 240 nm was used for data integration. Chromatograms were integrated using Agilent Chemstation Rev. B.03.01 software (Waldbronn, Germany). The total run time was five minutes.

### 2.4. Sample Processing and Estimation of Amiodarone

A liquid–liquid extraction method developed by Rodrigues et al. for monitoring amiodarone in rat plasma was validated for use in porcine serum [14]. Stock solutions of amiodarone and tamoxifen were prepared by accurately weighing and transferring a known quantity of each drug into separate volumetric flasks. Amiodarone was diluted in methanol and added to porcine serum (130 µL) so as to obtain concentrations of 1, 2, 3, 6, 12, 24, 48, and 96 µg/mL, and a final volume of 150 µL. Tamoxifen, the internal standard (ISTD), was prepared to a final concentration of 1 mg/mL, further diluted in methanol, and added to obtain a concentration of 25 µg/mL. Each standard concentration was spiked with 20 µL of the ISTD, followed by 630 µL of cold (4 °C) acetonitrile. This was then vortex-mixed for 1 min, centrifuged at 17,000 rpm for 15 min at 4 °C, and the supernatant transferred to a new tube. Then, 500 µL of n-hexane was added to this supernatant, vortex-mixed for 1 min, centrifuged at 17,000 rpm for 15 min at 4 °C, and the upper organic layer transferred to a new glass tube. The sample was then re-extracted two more times with 500 µL of n-hexane using this method, and the entire extract was evaporated (TurboVap LV evaporator, Biotage, Charlotte, NC) under a nitrogen stream at 60 °C. The residue was then reconstituted in 150 µL of methanol, transferred to vial inserts, and placed into amber-colored HPLC vials for analysis (Table 1).

### 2.5. Assay Validation

Blank pig serum was prepared as above and analyzed for the assessment of potential interferences due to endogenous substances. Calibration curves were determined using amiodarone concentrations between 2 and 96 µg/mL. These concentrations were utilized to allow for assessment of therapeutic concentrations without the need to dilute samples. Peak area ratios comparing amiodarone to tamoxifen were plotted against nominal concentrations of the solutions and least-squares linear regression was used to determine the predictive equation. Intraday assay precision was measured by analyzing at least three replicates of each concentration. Precision was represented as the percentage relative standard deviation (% RSD) by dividing the standard deviation of the measured concentrations from the mean measured concentration. Accuracy was expressed as the percentage recovery (% RE) and calculated by dividing the measured value by the nominal concentration multiplied by 100. The lower limit of quantitation was defined as the value that resulted in a % RSD less than 20% in the replicates [17].

### 2.6. Pharmacokinetic Methods: Noncompartmental

Noncompartmental pharmacokinetic analysis was performed in Pmetrics package version 1.5.2 (Los Angeles, CA, USA) in R 3.6.1 (R Foundation for Statistical Computing, Vienna, Austria) [18]. Measured concentrations were utilized to calculate the areas under the concentration time curve for 6 h (AUC6h) with trapezoidal approximation. Concentrations below the analytical lower limit of quantitation were censored so that the terminal elimination phase could be calculated from the final 3 decreasing concentrations.

### 2.7. Statistical Evaluation

Differences in pharmacokinetic parameters were compared between the control and IL group by utilizing the Wilcoxan–Mann–Whitney test in Stata version 15.1 (College Station, TX, USA). A *p*-value less than 0.05 was considered significant.

## 3. Results

### 3.1. Assay Method Validation

Among the various wavelengths studied, 240 nm displayed the highest UV absorbance for amiodarone (data not shown). Retention times of amiodarone and tamoxifen in serum were 2.0 min and 1.2 min, respectively. No significant interfering peaks from endogenous substances in blank serum were observed in drug-free porcine serum at the retention time of the analyte and the internal standard. The assay was linear from 2 to 96 µg/mL, and the mean regression equation for nominal amiodarone concentrations (i.e., “x”) to integrated area ratios (i.e., “y”) in spiked plasma was found to be y = 0.0654x − 0.0121 (r^2^ = 0.9993) and y = 0.0697x − 0.0081 (r^2^ = 0.9986). The lower limit of detection for amiodarone using this method was found to be 2.0 µg/mL.

### 3.2. Pharmacokinetic Results

A summary of the pharmacokinetic parameters between groups can be found in Table 2. The half-life of amiodarone was longer in the IL group (3.12 vs. 0.85 h, *p* = 0.01). The clearance was similarly (but not statistically) slower in the IL group (89.7 L/h vs. 403.5 L/h, *p* = 0.06), leading to a respective numerically higher AUC (84.6 vs. 62.9 µg/mL × 6 h, *p* = 0.08). While the latter two pharmacokinetic parameters were not statistically different, trends remained within this relatively constrained sample size (i.e., *n* = 7 per group). Other pharmacokinetic parameters such as maximum concentration (Cmax) and time of maximum concentration (Tmax) did not differ, demonstrating that the experimental model was similar for both groups at baseline.

## 4. Discussion

This is the first study to monitor serum amiodarone following IL infusion after an acute amiodarone overdose. The use of serum amiodarone concentrations as a surrogate marker of drug exposure has not been widely adopted in clinical practice; however, adverse effects have been documented with serum concentrations above 2.5 µg/mL in human and animal studies [9]. Hemodynamic adverse effects such as hypotension and prolongation of the atrioventricular conduction have been noted with the intravenous formulation, presumably as a function of acute overdose. These adverse effects are most commonly studied in rat models; however, swine may be a more appropriate model since they have a cardiovascular system more analogous to humans and have comparable responses to cardiac arrest and resuscitation, unlike other animal models [19,20]. The utilization of swine in these studies is highly justifiable, as they have proven to be an effective model in biomedical research and share a number of anatomical and physiological traits with humans. In particular, their cardiovascular system exhibits vascular and hemodynamic traits typical of most mammals and is highly translatable to human application [17,21,22]. Specifically, swine are an ideal model for acute amiodarone overdose studies as their response to anesthesia, cardiac arrest, and resuscitation is similar to that of humans, unlike rodents and rabbits [19,20,23].

Research so far has shown that the hypotensive response to amiodarone not only occurs during and shortly after administration of the loading dose but also is sustained during continuous infusion of a maintenance dose. This response is related both to the antiarrhythmic action of the drug and the polysorbate cosolvents (e.g., polysorbate 80 and benzyl alcohol) used to formulate intravenous amiodarone [24]. The initial period of hypotension may be due to decreases in both cardiac output and systemic vascular resistance, but the latter phenomenon may reverse after several minutes, leaving the direct inotropic effect as the primary reason for the hypotension [25].

In the parent study [13], amiodarone overdose caused significant hypotension accompanied by a significant bradycardia, while IL administration resulted in a short-term improvement in cardiac output, systolic, and mean arterial pressure. Furthermore, IL increased central venous pressure without a concomitant increase in right atrial diastolic pressure. Although this transient hemodynamic improvement was difficult to explain, the current pharmacokinetic findings shed more light on the foggy landscape of amiodarone toxicity and treatment.

First, our results strengthen the notion that IL administration alone may not prevent the adverse effects of amiodarone. In our swine, administration of IL did not appear to significantly increase clearance of amiodarone; however, trends toward longer blood residence were seen in the IL group. We did not measure tissue concentrations of amiodarone. It is possible that IL prevents tissue accumulation, which in turn would decrease adverse events. Ultimately, if increased amiodarone remains in the blood after IL administration, a subsequent directed removal (e.g., plasmapheresis) could eventually prove beneficial (tricyclic antidepressant overdose) [26]. Previously, it has been shown that the accumulation of amiodarone could trigger adverse effects in organs and tissues because of trafficking through late endosomes and induce a Niemann–Pick C-like phenotype [27]. However, it is unclear how this relates to our acute toxicity study and further investigations would be necessary to assess these types of adverse effects.

In our previous report, we found transient hemodynamic improvement in the IL group. This could have contributed to the differences in the composition of IL and saline and the slower amiodarone clearance observed in the IL group. The physical properties of IL could have acted as a volume-expanding colloid solution. Interestingly, IL may also improve hemorheological parameters, therefore improving blood flow properties at low shear forces [28]. One may argue that in a 75 kg human, the currently recommended dose of IL may be small compared to his total blood volume and, based on the Hagen–Poiseuille equations (R = 8 μL/πr4), this dose will only minimally increase flow rates. However, in our 19 ± 2 kg swine, IL administration may have exerted significant hemodynamic responses, although transient due to its effects on pharmacokinetic disposition of amiodarone, i.e., the decreased drug clearance that prolonged its action on the cardiovascular system. Further research with additional control groups, such as whole blood or colloids, may help to clarify this point.

Another important parameter for the interpretation of our findings is the constitution of IL, which is composed of soybean oil. The major unsaturated fatty acid in soybean oil triglycerides is linoleic acid, which is converted to arachidonic acid after intravenous administration [29]. The latter is further transformed to eicosanoids and thus thromboxane and leukotriene B4, which may exert vasoconstrictive effects. This is very important considering that capillary radii dominate the peripheral resistance and therefore blood flow, and IL may have improved systemic vascular resistance in our swine. Other possible mechanisms which may work in concert with the aforementioned effects of IL include fatty acid supply, reversal of mitochondrial dysfunction, inotropic effects, and inhibition of nitric oxide release [13,30].

The authors recognize several limitations in the interpretation of the present findings. Given the small experimental sample sizes in this study, the outliers caused IL infusion to seemingly lead to a statistically insignificant change in serum amiodarone levels. However, since amiodarone and its principal active metabolite, desethylamiodarone, are lipophilic, IL should theoretically capture the free amiodarone in blood. Thus, there is a need for a larger study that can elucidate any potential therapeutic benefits of IL infusion in this context. Despite the fact that IL showed significant improvement in the first minutes of infusion, this study did not record any benefit in the monitoring period. Larger studies are necessary for the full elucidation of the pleiotropic effects of IL.

## 5. Conclusions

In this pilot study, administration of IL did not appear to alter amiodarone clearance. However, the blood half-life of amiodarone was longer in the IL animals compared to the control group. Further studies are warranted to shed light on the complex pharmacokinetics of amiodarone.

## Figures and Tables

**Table 1:** Initial accuracy and precision (using liquid–liquid extraction).

Concentration (µg/mL)	Average of Area of Amiodarone to ISTD Area Ratio	SD	Accuracy (% RE)	Precision (% RSD)
1.765	0.1090	0.0196	104.83	17.9778
2.647	0.1388	0.0250	87.06	17.9866
5.294	0.2843	0.1359	85.54	47.8049
10.588	0.7692	0.0737	112.73	9.5766
21.176	1.3069	0.0708	95.16	5.4208
42.353	2.7902	0.0493	101.09	1.7667
84.706	5.5259	0.4027	99.89	7.2871

Abbreviations: ISTD, internal standard; SD, standard deviation; RE, percentage recovery; RSD, relative standard deviation.

**Table 2:** Pharmacokinetic parameters in a pig model with and without Intralipid rescue strategy.

	Intralipid					Control				
	Mean	SD	p50	p25	p75	Mean	SD	p50	p25	p75
AUC (μg/mL × 6 h)	84.61	48.69	76.50	52.64	130.17	62.89	103.87	21.52	9.08	69.53
Cmax (μg/mL)	196.35	188.94	94.90	41.65	454.56	279.99	462.19	86.43	32.95	368.36
Tmax (h)	0.21	0.04	0.25	0.17	0.25	0.18	0.09	0.17	0.08	0.25
CL (L/h)	89.65	65.69	62.30	41.96	123.40	403.46	364.81	280.91	108.07	792.41
VDss (L)	239.85	136.36	270.83	70.30	365.85	134.04	137.66	92.10	25.12	220.54
Thalf (h)	3.12	2.42	2.23	1.32	6.23	0.85	1.31	0.35	0.26	0.65

Abbreviations: SD, standard deviation; AUC, area under the curve; Cmax, maximum concentration; Tmax, time of maximum concentration; CL, clearance; VDss, volume of distribution at steady state; Thalf, half-life.

## Data Availability

The data presented in this study are available on request from the senior author.
